# Discovery and Biological Evaluation of CD147 *N*-Glycan Inhibitors: A New Direction in the Treatment of Tumor Metastasis

**DOI:** 10.3390/molecules26010033

**Published:** 2020-12-23

**Authors:** Wenqian Li, Daojiong Wang, Yushu Ge, Lei Zhang, Jiang Wu, Dan Liu

**Affiliations:** 1 Hefei National Laboratory for Physical Sciences at Microscale, The CAS Key Laboratory of Innate Immunity and Chronic Disease, School of Basic Medical Sciences, Division of Life Sciences and Medicine, University of Science and Technology of China, Hefei 230027, China; wenqianl@mail.ustc.edu.cn (W.L.); wangdj7@mail.ustc.edu.cn (D.W.); geyushu@ustc.edu.cn (Y.G.); 2 Department of Pharmacy, The First Affiliated Hospital of USTC, Division of Life Sciences and Medicine, University of Science and Technology of China, Hefei 230001, China; 76zhanglei@163.com

**Keywords:** CD147, *N*-linked glycosylation, inhibitors, metastasis

## Abstract

*N*-glycosylation is instrumental to the regulation of CD147 functions, including the maturation of CD147, secretion of matrix metalloproteinases (MMPs), and promotion of tumor metastasis. Glycosylated CD147 is highly expressed in various cancer types, participates in metastasis, and is associated with the poor prognosis of malignant tumors. However, to date, there has been little development of target-specific inhibitors for CD147 glycosylation. In this work, we report a strategy for discovering CD147 glycosylation inhibitors through computer-aided screening and inhibition assays. Four compounds were screened as potential CD147 glycosylation inhibitors. Of these, compound **72** was finally identified as the best candidate. Further experiments confirmed that compound **72** inhibited the production of MMPs and the metastasis of cancer cells in the Hela cell line. Results further suggest that compound **72** could promote the expression of E-cadherin by targeting CD147, thereby inhibiting tumor migration. Finally, the structures of the other potential CD147 *N*-glycosylation inhibitors may eventually provide guidance for future optimization.

## 1. Introduction

Cancer is expected to be the leading cause of death, and the incidence and mortality of cancer are increasing rapidly worldwide [1]. Activating invasion and metastasis is one of the hallmarks of cancer [2]. Previous studies have consistently demonstrated that metastasis is the leading cause of poor cancer prognosis [3,4,5]. Cervical cancer is known to be one of the most common cancers that cause female deaths in the world [1]. Metastatic cervical cancer can lead to poor prognosis [6]. As a result, the development of an anti-metastatic drug for better biomarkers has been urgently in need [7].

Glycosylation is a common post-translational modification of proteins [8]. *N*-linked oligosaccharides are located on the Asn of the NXS/T (Asn-X-Ser/Thr in which “X” is any amino acid except Pro) sequon [9] and participate in the biosynthesis, folding, transportation, and stability of membrane proteins [10]. Studies have found that nearly all cell membrane proteins in eukaryotes contain *N*-glycosylation modifications in the extracellular domain [11].

The CD147 membrane protein belongs to the immunoglobulin superfamily [12,13] and is actively involved in the invasion and migration of cancer cells [14,15,16,17]. Typically, it is highly expressed in many different types of human tumors [18,19,20], including cervical cancer [21,22], but at very low levels in normal cells [20,23]. CD147 plays a key role in the progression of cancer by stimulating the secretion of vascular endothelial growth factor (VEGF), MMPs, and cytokines. Furthermore, CD147 is involved in tumor cells adhesion, tumor angiogenesis, and multi-drug resistance [22,24,25]. Importantly, a high-level expression of glycosylated CD147 in tumors can effectively stimulate the expression of MMPs through neighboring fibroblasts and the tumor cells themselves and promote cancer metastasis [14,19,21,25]. Thus, these data identify CD147 as a tumor therapeutic biomarker.

Three *N*-linked glycosylation sites on CD147 were Asn44, Asn152, and Asn186. It is reported that glycosylation on CD147 asparagine residues is necessary for stabilizing CD147, transporting CD147 to the cell membrane, modulating the secretion of MMPs, and finally initiating extracellular matrix remodeling [26,27,28]. When CD147 protein glycosylation is inhibited, CD147 will misfold, accumulate in the endoplasmic reticulum (ER), and eventually enter ER-associated degradation (ERAD) [29,30]. In a recent study, Wan Huang et al. used mAbHAb18 to purify natural CD147 from patients’ lung cancer tissue specimens by immunoaffinity chromatography. The resulting *N*-glycan structure of CD147 was characterized by nanospray ionization mass spectrometry (NSI-MS). These results demonstrated that purified CD147 had both high mannose and dual antenna complex oligosaccharides, and it also contained core fucosylation (28.8%) and terminal sialylation modification, which is closely associated with tumor metastasis [30]. Summarily, glycosylated CD147 is an attractive drug target for preventing cancer metastasis.

Previous studies have focused on antibody drug discovery. For instance, a monoclonal antibody drug—Licartin [31], and a chimeric antibody—cHAb18 [32] targeting CD147, were launched by Chen’s group and applied in a clinical setting. However, the above antibodies contain immunogenicity or radioactivity, such as human anti-mouse antibody reaction (HAMA), thereby limiting large-scale clinical application [33]. More recently, research has focused on small molecular drugs for targeted cancer therapies. To our knowledge, researchers have so far identified only one small synthetic compound, AC-73, that targets CD147 dimerization [33]. Only a few studies have reported CD147-selective glycosylation inhibitors. The known *N*-glycosylation inhibitors include tunicamycin, swainsonine, and 3′-azidothymidine [34,35]. However, these are not selective, so there remains a need to discover novel small compounds targeting CD147 glycosylation.

Current means of glycosylation detection are relatively out-of-date [27], so high-throughput screening cannot be conducted. In order to obtain novel selective glycosylation inhibitors of CD147, virtual screening from a drug-like compound bank was carried out. The most significant candidates identified by molecular docking were further evaluated by Western blot analysis.

Post-translational modifications of proteins include phosphorylation, acetylation, methylation, ubiquitination, and glycosylation; all of which cause changes to the protein molecular weights. Of these, changes associated with glycosylation can be more clearly observed in the electrophoretic mobility than the other modifications. For instance, the unglycosylated CD147 core protein has a molecular weight of 27 kDa, while the molecular weight of the glycosylated form is approximately between 43 and 66 kDa. Western blot results show that glycosylated CD147 has two bands: HG-CD147 (Highly glycosylated CD147) is approximately 40–60 kDa and LG-CD147 (Lowly glycosylated CD147) is about 32 kDa. Based on previous studies, changes in CD147 due to glycosylation reduce CD147 electrophoretic mobility in Western blot analysis, as was observed in this study [28,35,36].

The purpose of this study was to explore the potent and selective CD147 glycosylation inhibitors and identify structures representative of an effective glycosylation inhibitor. Additionally, biological activities of these inhibitors were tested, including cell viability, motility, and invasion. We are confident that the novel inhibitors targeting glycosylation will become extraordinary drug-like inhibitors, which will greatly improve the prognosis of cancer patients.

## 2. Results

### 2.1. Virtual Screening and Hit Validation

**Figure 1:**
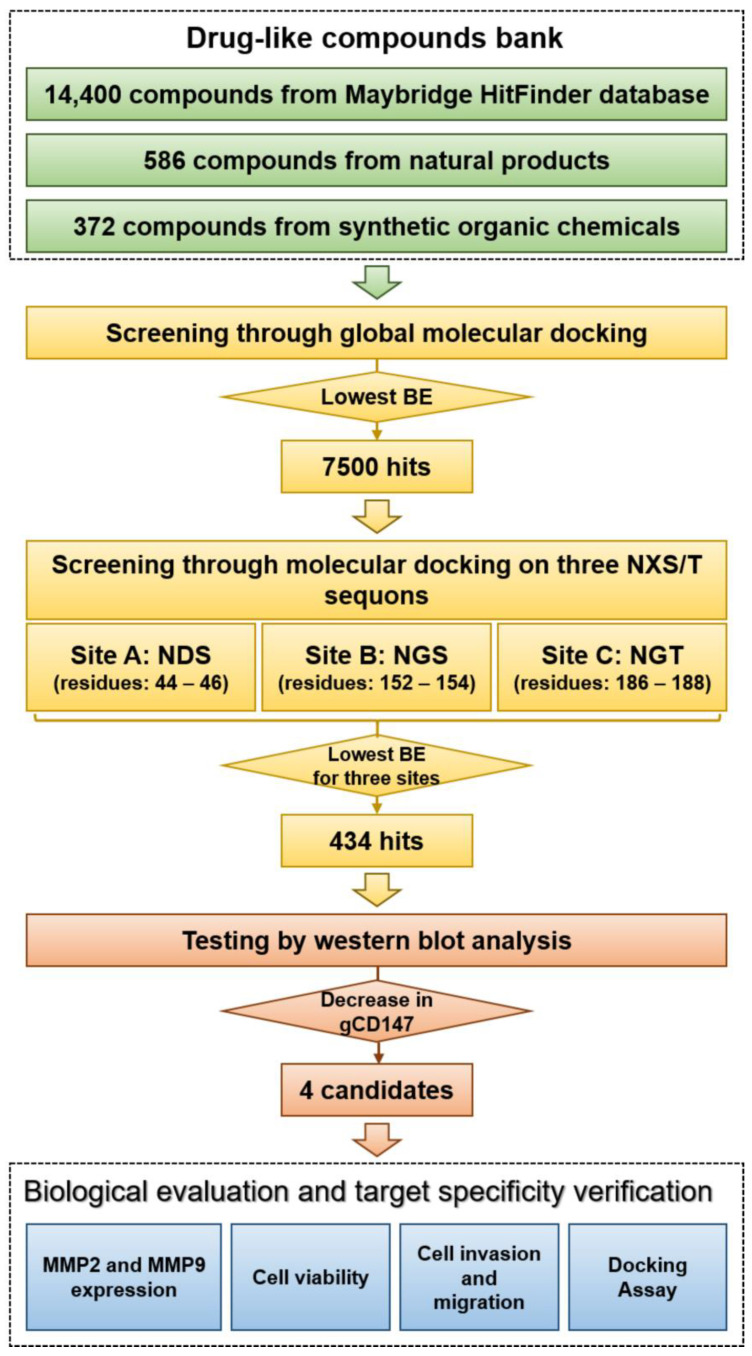
Workflow of the experimental screening of CD147 glycosylation inhibitors. BE: binding energy; gCD147: glycosylated CD147.

Natural products isolated from animals, plants, and microorganisms, due to their diversity, are important sources of new drug candidates to treat inflammation, cardiovascular diseases, and cancers [37,38]. Synthetic organic chemistry assumes a major part in drug improvement and development over the globe. Some chemical structures are essential for bioactivity in natural products and pharmacophores in drug discovery such as spiroketal cores. Therefore, synthetic organic compounds will also become potential new drugs. For this study, a drug-like bank of compounds including natural products, synthetic organic chemicals, and commercially obtained compounds was established as candidate drug sources (Figure 1).

The workflow of the experimental screening study is presented in Figure 1. Over 15,000 compounds from drug-like compounds bank were screened in silico. Two layers were conducted for virtual screening. Global docking was used to perform as a rough docking experiment for the first layer and resulted in 7500 hits. These 7500 hits were docked with the sites on the three NXS/T sequons of CD147 as the second layer. Normally, the lower binding energy indicates better binding affinity with the search area. A total of four hundred thirty-four hits from the library were selected for biological testing after virtual screening. Hela cells were co-cultured with these compounds respectively and then the cell lysates were analyzed by Western blot (Figures S1–S7 in Supplementary Materials).

**Figure 2:**
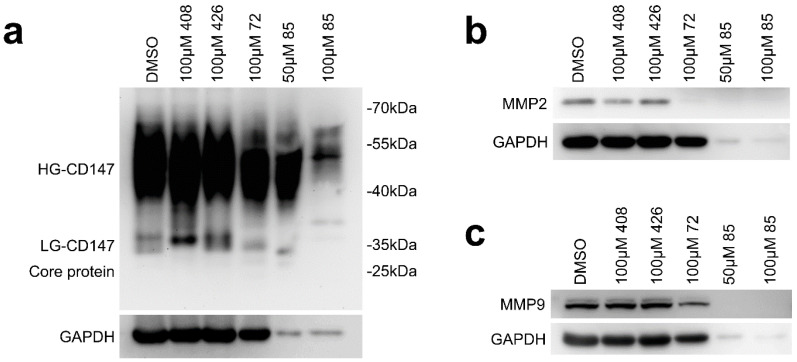
Four candidate inhibitorswere identified following docking studies and then Western blot analysis in Hela. (**a**) The effects of compounds **72**, **85**, **408**, and **426** on reducing of CD147 glycosylation for 24 h (compounds **72** and **85** for 12 h). (**b**) The effect of four compounds on matrix metalloproteinase (MMP2) expression and (**c**) MMP9 expression for 24 h (compounds **72** and **85** for 12 h).

Four candidates (Table 1) that caused significant changes in molecular weight of CD147 were initially identified for further biological evaluation (Figure 2). After treatment with 100 μM of compound **72**, the amount of HG-CD147 and LG-CD147 was reduced compared with the DMSO group and the weak abnormal band of CD147 in 27–30 kDa could be observed, which reflected the damage of glycosylation. Similar results were also obtained under the treatment of compound **85** at the concentration of 50 μM, and the expression of the reference gene GAPDH was also significantly reduced. A significant decrease in HG-CD147 was observed when cells were treated with compound **85** at the concentration of 100 μM. At the same time, the band of LG-CD147 almost disappeared. Compound **408** slightly down-regulated the expression of LG-CD147, which made change of molecular weight of LG-CD147. Compound **426** slightly impaired the glycosylation of CD147, including the reduction of HG-CD147 and the presence of the weak band between 27 and 30 kDa (Figure 2a). Of these, compounds **72**, **85**, and **408** are synthetic organic chemicals [39,40]. Compound **426** was commercially obtained from Maybridge HitFinder. Taken together, the studies described above suggest that compound **72** significantly destroyed the glycosylation of CD147 among the four compounds.

### 2.2. Effect of Candidate Inhibitors on MMPs Expression

As previously noted, CD147 glycosylation induces MMPs secretion of tumor cells [41,42]. Preventing CD147 glycosylation can lead to a lower expression of MMP2 and MMP9, which is essential in tissue remodeling and cancer metastasis [25,30]. The effects of the four candidate inhibitors on MMP2/MMP9 expression were analyzed (Figure 2b,c). The expression of both MMP2 and MMP9 were obviously reduced compared with the DMSO group after cells were treated with 100 μM of compound **72**, and the expression of MMP2 almost disappeared. For compound **85**, the expression of MMP2 and MMP9 was significantly reduced, and similar results also appeared on the reference gene GAPDH. For compound **408**, it reduced the expression of MMP2. There was no change to MMP2 and MMP9 after treatment of compound **426**. These studies demonstrate that compound **72** could impair the glycosylation and further reduce the expression of MMP2 and MMP9.

### 2.3. Cytotoxicity Test of Candidate Inhibitors

**Figure 3:**
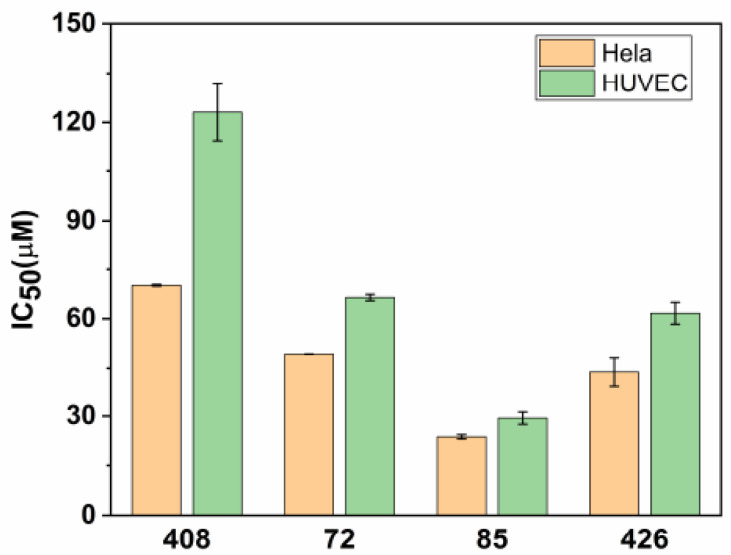
Half inhibitory concentration (IC_50_) values of compounds **72**, **85**, **408**, and **426** for Hela and HUVEC (human umbilical vein endothelial cells) cell lines (mean ± SEM, standard error of mean was calculated with three replicates).

Half inhibitory concentration (IC_50_) is widely used to evaluate cytotoxicity [43]. Hela and HUVEC (human umbilical vein endothelial cells) cell lines were used for testing the cytotoxicity of the four candidate inhibitors (Figure 3). Of these, the IC_50_ of compound **408** was higher than those of the other compounds in different testing cell lines. For compound **408**, the IC_50_ values were 70.13 ± 0.25 μM in Hela and 123.05 ± 8.61 μM in HUVEC. For compound **72**, the IC_50_ values were 49.24 ± 0.04 μM in Hela and 66.43 ± 0.96 μM in HUVEC. The IC_50_ values of Hela were much lower than those of HUVEC, which indicated that these compounds were much more toxic to Hela than to HUVEC. Notably, compound **408** exhibited the lowest cytotoxicity for Hela and HUVEC, which was followed by compounds **72**, **426**, and **85**.

### 2.4. Effect of Candidate Inhibitors on Tumor Cell Invasion and Migration

Metastasis is the main obstacle to cancer therapy. CD147 can promote tumor metastasis, and this function is closely related to glycosylation according to previous studies [36,44].

Therefore, in this study, the four selected candidates were evaluated for their ability to inhibit cell invasion and migration by transwell assay in vitro. Transwell membranes coated with Matrigel that serve as a reconstituted basement membrane in vitro were used to evaluate cell invasion (non-invasive cells were blocked from migrating through the pores of the membrane by Matrgel, and only invasive cells can degrade the Matrigel and invade through the pores), while membranes uncoated with Matrigel were used to study cell mobility. The work concentrations were set below the half IC_50_ value as far as possible to prevent the cytotoxicity and ensure the active concentration of these compounds.

**Figure 4:**
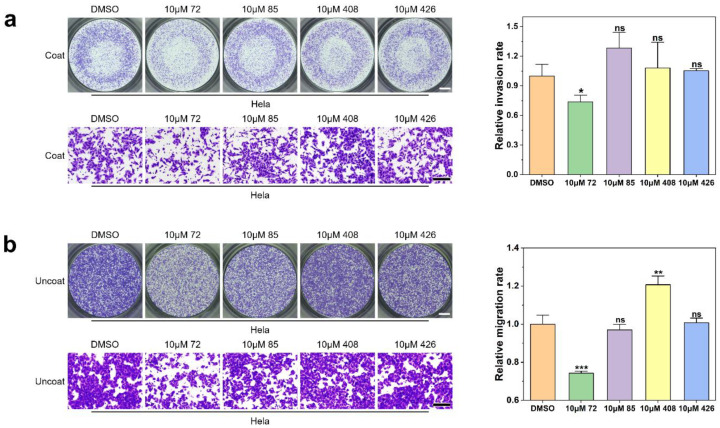
The effect of compound **72**, **85**, **408**, and **426** on Hela cell invasion and migration. (**a**) Invasion ability of Hela cells after treatment with different compounds for 24 h. Scale bars:1000 μm (upper left) and 200 μm (lower left). (mean ± SD, standard deviation was calculated with three replicates). (**b**) Migration ability of Hela cells after treatment with different compounds for 24 h. Scale bars: 1000 μm (upper left) and 200 μm (lower left). (mean ± SD, standard deviation was calculated with three replicates). * *p* < 0.05, ** *p* < 0.01, *** *p* < 0.001, ns indicates *p* > 0.05.

Results of the transwell assay with coated chambers indicated that only compound **72** exhibited inhibitory activity on cell invasion (Figure 4a). After treatment with 10 μM of compound **72**, the number of cells passing through the pores in the transwell membrane has been significantly reduced compared with DMSO group (decreased by 26.30% ± 6.79%). Compounds **85**, **408**, and **426** had no significant effect on cell invasion ability.

The results of transwell assay with uncoated chambers showed that when Hela cells were treated with 10 μM of each compound **72**, **85**, **408**, and **426** for 24 h, cell migration was inhibited by 25.73% ± 1.01%, 3.03% ± 2.94%, −20.69% ± 4.58%, and −0.69% ± 2.51% compared with the control cells, respectively (a negative value means that migration was facilitated). Here, only compound **72** inhibited the mobility of Hela cells significantly (Figure 4b). Surprisingly, compound **408** promoted the migration of Hela cells, and the research on compound **408** is still ongoing.

Collectively, the results of these studies indicated that both the invasion and migration of Hela cells were inhibited by 10 μM concentration of compound **72**.

### 2.5. Target Validation of Compound **72**

From the above results, it is interesting to explore whether compound **72** could inhibit the invasion and migration of malignant cells by targeting CD147.

To confirm whether the compounds specifically target CD147, a stable cell line that knocked out CD147 was established (Figure S8).

**Figure 5:**
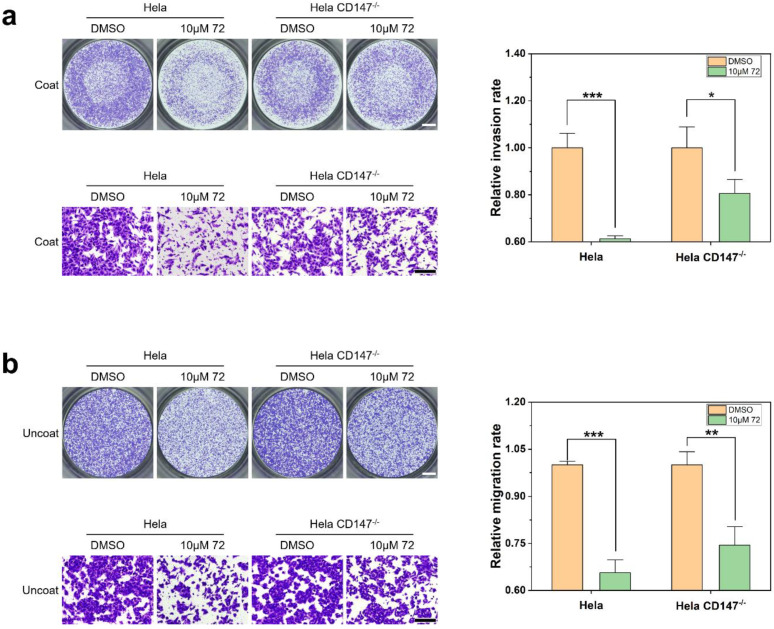
In vitro invasion and migration of Hela and Hela CD147 knockout cells treated with compound **72** for 24 h. (**a**) Effect of compound **72** on cell invasion in Hela and Hela CD147 knockout cells. Scale bars: 1000 μm (upper left) and 200 μm (lower left) (mean ± SD, standard deviation was calculated with three replicates). (**b**) Effect of compound **72** on cell migration in Hela and Hela CD147 knockout cells. Scale bars: 1000 μm (upper left) and 200 μm (lower left) (mean ± SD, standard deviation was calculated with three replicates). * *p* < 0.05, ** *p* < 0.01, *** *p* < 0.001.

In terms of cell invasion ability, the invasion rate was 61.33% ± 1.29% (Figure 5a) after cells were treated with 10 μM compound **72**, and cell invasion ability was inhibited significantly compared with control group, while the invasion rate of CD147 knockout Hela cells was 80.63% ± 5.85% (Figure 5a).

In terms of cell migration ability, the migration rate was 65.63% ± 4.14% (Figure 5b) after treatment with 10 μM compound **72**, and cell migration ability was inhibited significantly compared with the control group, while the migration rate of CD147 knockout Hela cells was 74.46% ± 5.90% (Figure 5b).

These results suggest that Hela cells were more sensitive to compound **72** than CD147 knockout Hela cells. The inhibition rate of compound **72** on CD147 knockout Hela cells was not as good as on Hela, which indicated that to some extent, compound **72** inhibits the invasion and migration of malignant cells by targeting CD147. To sum up, we speculate that compound **72** can inhibit the glycosylation of CD147, further inhibiting the secretion of MMPs and cell metastasis in Hela.

### 2.6. Effect of Compound **72** on Tumor-Associated Molecules by Targeting CD147

**Figure 6:**
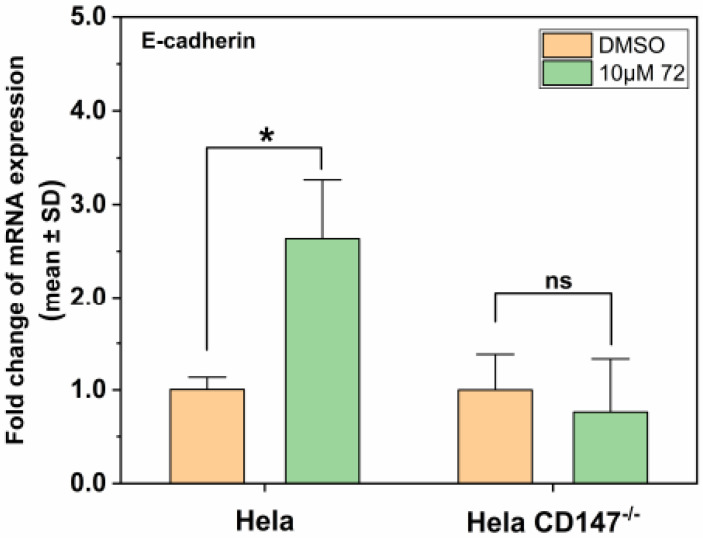
Effect of compound **72** on the mRNA expression of E-cadherin in Hela and Hela CD147 knockout cells (mean ± SD, standard deviation was calculated with three replicates). * *p* < 0.05, ns indicates *p* > 0.05.

Epithelial-to-mesenchymal transition (EMT) plays an important role in cancer metastasis [45,46]. E-cadherin is a prototypical marker for the transition of epithelial (E) cells to cells with a mesenchymal (M) phenotype [45]. Loss of the key adhesion molecule E-cadherin in carcinoma cells is the best-characterized alteration indicative of cancer [2]. Through the above experiments, we wondered whether compound **72** affected tumor metastasis-related molecules through CD147, thereby controlling the metastasis of Hela cells. The results of this study demonstrated that 10 μM of compound **72** promoted E-cadherin expression in Hela but not in CD147 KO Hela. That is, compound **72** promoted mRNA expression of E-cadherin by targeting CD147 (Figure 6).

### 2.7. Computational Characterization of the Binding Modes between Compound **72** and CD147 Glycosylation Sites

**Figure 7:**
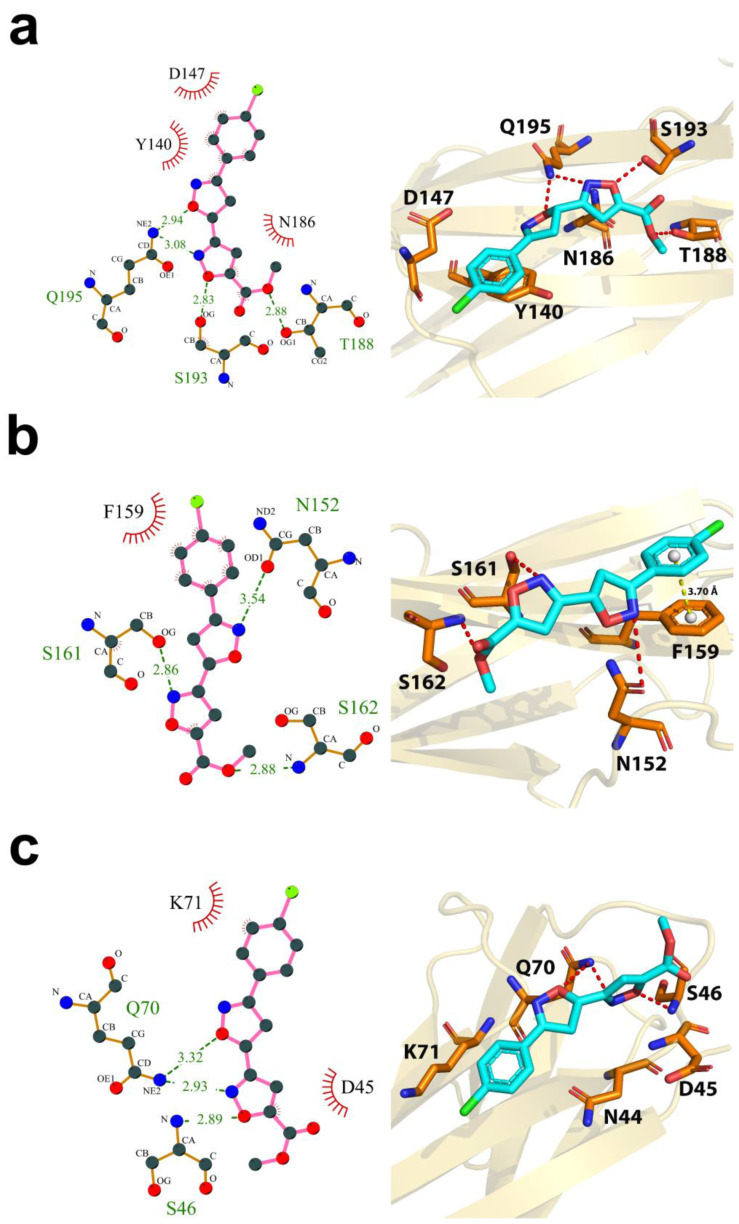
Docking of compound **72** on the glycosylation sites (**a**) N186, (**b**) N152, and (**c**) N44 of CD147. The left panels are LIGPLOT diagrams. Dark slate gray ball, carbon; blue ball, nitrogen; red ball, oxygen; lime green ball, chlorine. Hydrogen bonds are showed as dotted olive green lines. The right panels are binding modes between compound **72** and residues on glycosylation sites. CD147 is shown as ribbons; key residues (orange) and compound **72** (blue) are depicted as sticks; hydrogen bonds are shown as red dashes; the centroids of aromatic rings are represented as gray balls.

Binding modes of the active compound with three different glycosylation sites of CD147 were studied by molecular docking experiments. The binding energy of compound **72** on site N186, N152, and N44 was predicted as −5.6, −5.4, and −5.1 kcal/mol, respectively (Figure 7). Since a lower binding energy reflects greater binding affinity between the compound and the binding site, compound **72** may be more inclined to bind on site N186. For compound **72**, hydrogen bonds formed with T188, S193, and Q195 contribute the most for interacting with CD147. Moreover, the hydrophobic interactions formed between compound **72** and residues N186, Y140, and D147 strengthen the protein-inhibitor interaction (Figure 7a). Together, it is noticeable that the conserved acceptor sequence—NXS/T (N186, G187, and T188) in *N*-glycosylation was completely blocked by compound **72**, so the unexposed N186 residue may not be glycosylated by glycosyltransferases. In addition, compound **72** may also interact with site N152 and N44 with higher binding energy, which indicates weaker binding affinity (Figure 7b,c). Notably, besides hydrogen bonding interactions and hydrophobic contacts, the aromatic ring of compound **72** also formed π stacking with the phenyl ring of F159, which may enhance inhibitory activity (Figure 7b).

## 3. Discussion

As previous research reported, metastasis is the leading cause of death for cancer patients. Therefore, one of the central problems in the anti-tumor field is anti-metastasis. In the past, predominant cancer treatment focuses on cancer proliferation, rather than metastasis, so that the overall survival rate of patients with metastatic cancer has not improved significantly [4]. Although there are a lot of research studies on the development of anti-cancer drug, cancer treatment has always lacked effective anti-metastatic drugs [47]. The glycosylation of CD147 is closely related to tumor metastasis, including facilitating the mobility of malignant cells, promoting the secretion of MMPs, further leading to invasion of tumor cells, and so on. Importantly, CD147 is highly expressed in tumor cells rather than normal cells [20,23]. Therefore, CD147 is an ideal cancer target for metastasis.

In this work, based on virtual screening, we attempted to identify a series of potential inhibitors targeting CD147 glycosylation from the drug-like compounds bank. A small-molecule compound **72** that produced the inhibitory activity to CD147 glycosylation was identified, and its biological activity has not been discovered before [40]. Here, results demonstrated that compound **72** exhibited the highest inhibition potential for MMPs and furthered tumor metastasis among the candidate inhibitors. In addition, target validation suggested that compound **72** may reduce the metastasis of malignant cells depending on targeting CD147. On the other hand, it is interesting to note that compound **72** could affect cancer metastasis, to a certain degree, through EMT-related molecules—E-cadherin by targeting CD147.

To our knowledge, *N*-glycosylation inhibitors include tunicamycin (TM), swainsonine (SW), 3′-azidothymidine (AZT), castanospermine (CSP) and deoxymannojirimycin (DMJ), which induce the universal inhibition of intracellular glycosylation. Among them, 3′-azidothymidine (AZT) was reported to inhibit CD147 glycosylation at the 230 μM and 460 μM, but the research focused on the effect of glycosylation inhibition for cell proliferation. The crucial function of anti-metastasis has not been analyzed. However, AZT was not obtained by targeting CD147, and its selectivity has not been studied [35]. Although CD147 glycosylation is an important target for anti-metastasis, there are a few reports on CD147 glycosylation inhibitors. In the present study, CD147 structure-based virtual screening was employed to discover potential CD147 glycosylation inhibitors, and target validation was confirmed. Importantly, this research concentrated on anti-metastasis, and compound **72** showed a good inhibition of cell migration and invasion.

The NXS/T sequon is a unique acceptor sequence conserved in *N*-glycosylation [9]. The oligosaccharyltransferase recognizes the NXS/T sequon and transfers the oligosaccharide—Glc_3_Man_9_GlcNAc_2_ to a protein [48] Taken together, the above prediction results of the docking study suggested that compound **72** might potentially interact with residues on the three glycosylation sites to block the transfer of glycans to Asn in the NXS/T sequon. Considering the binding energy and interaction mode, compound **72** may preferred to bind on site N186. Moreover, due to the little difference in the binding energy of the three sites, the possibility of compound **72** binding on the other two sites should not be ignored.

Indeed, as the best candidate inhibitor, compound **72** needs to be modified in order to increase the activity and ensure the safety in future studies. First, there was little difference in cytotoxicity between normal cells and cancer cells. The selective cytotoxicity of the compounds still remains a significant challenge. Second, the biological activities will be tested in multiple CD147 overexpressed cancer cells. Third, the predicted binding surface between compound **72** and CD147 should be further explored to clarify the inhibitory activity of compound **72** to CD147.

Together, compound **72** may be a potential antineoplastic lead compound by reducing the glycosylation levels of HG-CD147 and further inhibiting the metastasis of tumor when used alone or in combination with other therapies. The selectivity to CD147 of compound **72** was not so ideal and still needs to be further improved. Structures of the four candidate inhibitors may represent potential new scaffolds of glycosylation inhibitors that deserve further studies (Table 1).

In conclusion, this research identifies the backbones of a potential CD147 glycosylation inhibitor and reveals a promising strategy for the treatment of metastatic cancer.

## 4. Materials and Methods

### 4.1. Materials

Hela and HUVEC were provided by The National Infrastructure of Cell Line Resource. Cell culture medium DMEM and fetal bovine serum were purchased from Biological Industries (BI, Beit HaEmek, Israel). Primers and penicillin/streptomycin were obtained from Sangon Biotech (Shanghai, China). Phanta max super-fidelity DNA polymerase was acquired from Vazyme Biotech (Nanjing, China). The cell culture inserts with 8.0 μm PET membrane and 24-well companion plates were bought from Corning (Corning, NY, USA). A CRISPR nuclease vector kit and T7 endonuclease Ι were individually purchased from Thermo Fisher Scientific (Waltham, MA, USA) and New England Biolabs (NEB, Ipswich, MA, USA). CD147 and MMP2 antibody was bought from Abcam (Cambridge, UK). MMP9 and GAPDH antibody was obtained from Proteintech (Wuhan, China). CCK-8 reagent was purchased from Biosharp (Hefei, China).

### 4.2. Compounds

The drug-like compounds bank consists of over 15,000 compounds (Figure 1). Compounds in the bank were renumbered using Arabic numerals. Part of the structure can be seen in Table 1. The compounds were dissolved in DMSO and then diluted in DMEM medium with 10% FBS to the working concentration. The final DMSO concentration was no more than 1% for all experiments. The four compounds mentioned in this study are compound **72** (methyl 3′-(4-chlorophenyl)-4′,5′-dihydro-[3,5′-biisoxazole]-5-carboxylate), compound **85** ((3a*S*,7a*R*)-7-(hydroxyimino)-2-phenyl-3a,4,5,6,7,7a-hexahydro-3H-indole 1-oxide), compound **408** ((2*S*,3*S*,4′*S*)-3-hydroxy-4′,6′-diphenyl-3′,4′-dihydro-3*H*-spiro[benzofuran-2,2′-pyran]-5′-carbonitrile), and compound **426** (1-(1*H*-indol-1-yl)-3-((2-((3-(trifluoromethyl)pyridin-2-yl)amino)ethyl)amino)propan-2-ol).

### 4.3. Cell Culture

All the cells were grown in DMEM medium supplemented with 10% fetal bovine serum and 1% penicillin/streptomycin at 37 °C in a humidified atmosphere that contained 5% CO_2_.

### 4.4. CRIPSR–Cas9 Genome Editing

The GeneArt CRISPR nuclease vector was provided by Thermo Fisher Scientific. The insert DNA fragments that code for four different target-specific guide RNA were designed at https://www.thermofisher.com. The sequence details can be seen in Table 2. Four CRISPR nuclease vectors were constructed and were transfected into the Hela cell line by Lipofectamine 3000. T7 Endonuclease I (T7E1) brought from NEB was used to detect CRISPR/Cas9 cleavage efficiency. Construction of the CD147 knockout Hela cell line proceeded according to instructions of the GeneArt^®^ CRISPR nuclease vector kit.

### 4.5. CCK-8 Cell Cytotoxity Assay

A Cell counting kit-8 (CCK-8) was used to evaluate the effect of candidate inhibitors on cell viability. Cells were seeded in 96-well plates with 100 μL media and incubated overnight. All solutions of candidate compounds were prepared in several different concentrations using the serial dilution method. Next, after treatment with diluted compound solutions for 24 h, 10 μL of CCK-8 reagent was added to each well and incubated at 37 °C for 1–4 h. The plates were read on a microplate reader at 450 nm wavelength.

The cell viability was calculated by Equation (1) [49].

(1) $$\mathrm{Cell}\;\mathrm{Viability}=\frac{{\mathrm{OD}}_{\mathrm{treated}}-{\mathrm{OD}}_{\mathrm{DMEM}}}{{\mathrm{OD}}_{\mathrm{control}}-{\mathrm{OD}}_{\mathrm{DMEM}}}\times 100\%$$

Here, OD_control_ is the absorbance value of the control group, OD_treated_ is the absorbance value of the test group, and OD_DMEM_ is the absorbance value of the cell blank group.

The half inhibition concentration (IC_50_ value) of compounds was calculated by cell viability using Logistic regression equation in a nonlinear curve fit of Origin software.

### 4.6. Transwell Assay

The transwell system, Corning cell culture chambers, was used to study cell invasion and migration of Hela in vitro. Cell culture inserts coated by Matrigel were used to study the cell invasion of malignant cells. Uncoated cell culture inserts were used to assess the migration property of malignant cells. The coated chambers were rehydrated for 2 h in humidified tissue culture incubator, 37 °C, 5% CO_2_ atmosphere before used. First, 750 μL DMEM containing 10% FBS was added to the lower chamber. Then, 500 μL of serum-free media cell suspension containing 3 × 10^5^ cells (for uncoated inserts) and 4.5 × 10^5^ cells (for coated inserts) were added to the upper chambers. The same concentrations of candidate compounds were added to both upper and lower chambers, respectively. After incubating at 37 °C for 24 h, non-invading cells were removed from the upper surface of the membrane, and then, the lower surface of the membrane containing invading cells was stained with crystal violet. The counting of invading cells was facilitated by photographing the membrane through the optical microscope. Image J software was used to analyze the photographs. Relative invasion rates were calculated by Equation (2), and relative migration rates were calculated by Equation (3), which were provided by the manufacturer.

(2) $$\mathrm{Relative}\;\mathrm{invasion}\;\mathrm{rate}=\frac{\mathrm{mean}\;\mathrm{of}\;\mathrm{cells}\;\mathrm{invading}\;\mathrm{through}\;\mathrm{each}\;\mathrm{drug}\;\mathrm{group}\;\mathrm{insert}\;\mathrm{membrane}}{\mathrm{mean}\;\mathrm{of}\;\mathrm{cells}\;\mathrm{invading}\;\mathrm{through}\;\mathrm{each}\;\mathrm{control}\;\mathrm{group}\;\mathrm{insert}\;\mathrm{membrane}}$$

(3) $$\mathrm{Relative}\;\mathrm{migration}\;\mathrm{rate}=\frac{\mathrm{mean}\;\mathrm{of}\;\mathrm{cells}\;\mathrm{migrating}\;\mathrm{through}\;\mathrm{each}\;\mathrm{drug}\;\mathrm{group}\;\mathrm{insert}\;\mathrm{membrane}}{\mathrm{mean}\;\mathrm{of}\;\mathrm{cells}\;\mathrm{migrating}\;\mathrm{through}\;\mathrm{each}\;\mathrm{control}\;\mathrm{group}\;\mathrm{insert}\;\mathrm{membrane}}$$

### 4.7. Virtual Screening and Molecular Docking

The crystal structure of CD147 was obtained from RCSB Protein Database Bank (PDB code: 3B5H, chain A). The structure of the biopolymer was analyzed and prepared for the virtual screening. Hydrogen atoms were added, and the biopolymer was charged using the AMBER FF99 method. A global docking study between all the compounds in the drug-like compounds bank and CD147 was conducted by AutoDock Vina software (version 1.1.2) [50] as the first layer of virtual screening. The side length of the cubic grid box was set as 60 angstroms centered on CD147. The exhaustiveness value was set to 8. The top 7500 hits with the lowest binding energy obtained from global docking were used in the second layer of virtual screening. Three docking sites were defined as the cubic grid boxes with a side length of 26 angstroms centered on the Cα atom of residue X in three NXS/T sequons of CD147, respectively. The exhaustiveness value was set to 8. Of all three sites, the binding mode with the lowest binding energy was recommended for each compound. All the compounds were ranked by binding energy, and the top 434 hits were selected for the following experiments.

After biological evaluation, molecular docking was employed to search for the optimal binding mode between the best candidate inhibitor (compound **72**) and CD147. A docking study was conducted three times in parallel by AutoDock Vina software [50] with refined parameters. The side length of cubic grid box was set to 18 and centered on three NXS/T sequons in order to cover the glycosylation sites and make compounds move freely for docking processing. The exhaustiveness value was set to 20. Structural analysis was performed with PyMOL. The results of the binding mode were analyzed by LigPlot+ program [51].

### 4.8. Western Blot Analysis

Cells were washed by PBS and later were lysed on ice by cell lysis buffer. Then, lysed cells were scraped. The cell lysates were centrifuged at 13,000 rpm at 4 °C for 8 min to remove cell debris. Protein concentration was determined by a BCA Protein Assay Kit to ensure that equal amounts of proteins were separated on the 10% SDS-PAGE and transferred to a PVDF membrane. After blocking by 5% BSA or nonfat milk for 1 h at room temperature, membranes were incubated with primary antibody overnight at 4 °C. To remove the unbound antibody, the membranes were washed 3× with TBST. Then, membranes were incubated with HRP-conjugated secondary antibody at room temperature for 2 h. Finally, the membrane was washed by TBST again and incubated with Vazyme ECL working buffer for photographing.

### 4.9. RNA Extraction, Reverse Transcription, and Real-Time Quantitative Polymerase Chain Reaction (qPCR)

Total RNA was extracted by TRIzol Reagent (from Thermo Fisher). Reverse transcription was performed with a cDNA synthesis kit (from Vazyme). All the operations were performed in an RNase-free environment. The human E-cadherin forward qPCR primer is 5′-ATGAGTGTCCCCCGGTATCTT-3′ and reverse primer is 5′-TCAGCCGCTTTCAGATTTTCA-3′. The human HPRT forward qPCR primer is 5′- GCTATAAATTCTTTGCTGACCTGCTG-3′ and reverse primer is 5′-AATTACTTTTATGTCCCCTGTTGACTGG-3′. All primers were synthesized by Anhui General Biol. Real-time PCR was performed with AceQ qRCR SYBR Green Master Mix (from Vazyme). All the data analyses were performed using the delta-delta Ct method (2^−∆∆Ct^ method). Results were normalized to the hypoxanthine phosphoribosyltransferase (HPRT) housekeeping gene as an internal control.

### 4.10. Statistical Analysis

Data were analyzed by Excel 2016 and OriginPro 2020. The results were expressed as mean ± standard error of the mean (SEM) or standard deviation (SD). Student’s *t*-test was used to evaluate significance between two independent groups (two tailed). Statistical significance was set at *p* value < 0.05 (* *p* < 0.05, ** *p* < 0.01, *** *p* < 0.001). ns indicates *p* > 0.05.

## Figures and Tables

**Table 1:** Detailed information of four candidatesfrom the compound bank.

Reference Number	Chemical Structure	MW (Da)
**72**	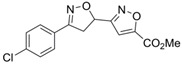	306.70
**85**	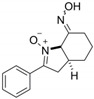	244.29
**408**	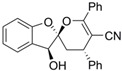	381.43
**426**	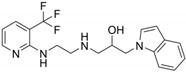	378.40

**Table 2:** Design single-stranded DNA oligonucleotides.

No.	Sequences(5′ to 3′)
1	CATCTCCATCGACACGCTCG
2	TCATGAACGGCTCCGAGAGC
3	CGTCAGAACACATCAACGAG
4	GTCGTCAGAACACATCAACG

## Data Availability

The data presented in this study are available from the corresponding author upon request.

## Supplementary Materials

The following are available online, Figures S1–S6: The preliminary screening of glycosylation inhibitors; Figure S7: A repeat of glycosylation inhibition assays for the potential compounds, Figure S8: Construction of CD147 knockout Hela cell line.

## Sample Availability

Samples of the compounds are not available from the authors.
