# Supplementary material for: Discovery and Biological Evaluation of CD147 N-Glycan Inhibitors: A New Direction in the Treatment of Tumor Metastasis
Source: Molecules. 2020 Dec 23;26(1):33. doi: 10.3390/molecules26010033 (PMC7794696; doi:10.3390/molecules26010033)
Supplement: Supplementary file 1 [file molecules-26-00033-s001.pdf]

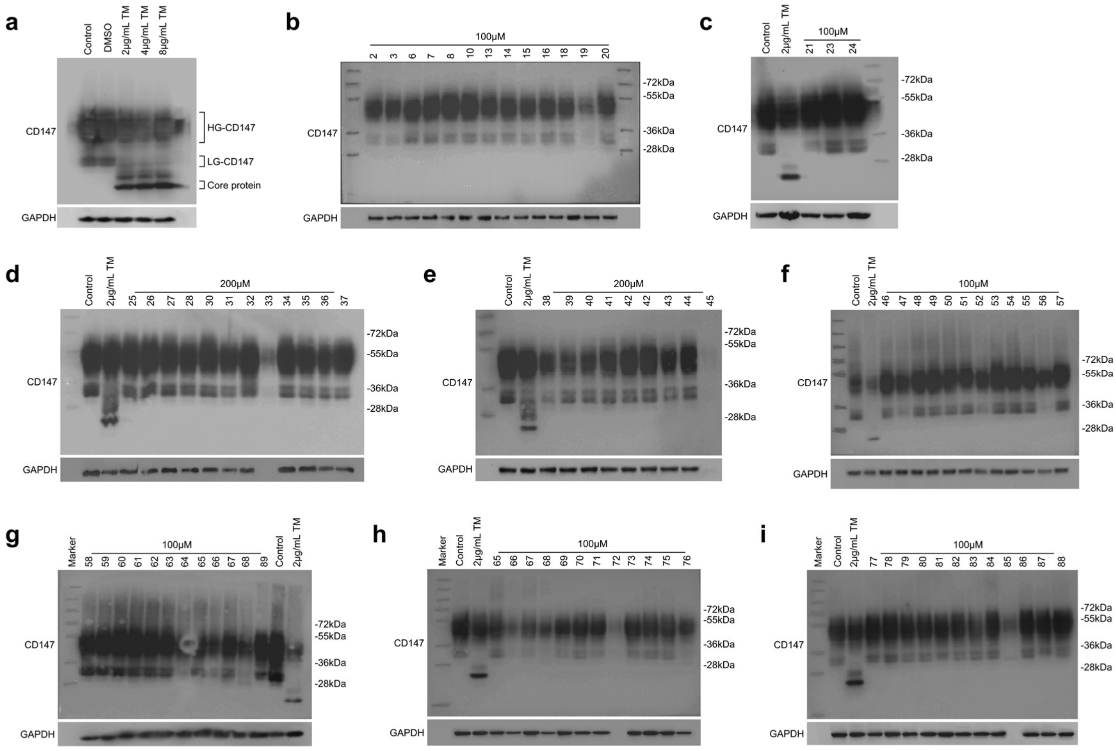

Figure S1. The preliminary screening of glycosylation inhibitors. **a**) Tunicamycin with varying concentrations inhibited CD147 glycosylation. **b**) Effect of compounds 2, 3, 6-8, 10, 13-16, and 18-20 on CD147 glycosylation in Hela for 24hr. **c**) Effect of compounds 21, 23, and 24 on CD147 glycosylation in Hela for 24hr. **d**) Effect of compounds 25-28 and 30-37 on CD147 glycosylation in Hela for 24hr. **e**) Effect of compounds 38-45 on CD147 glycosylation in Hela for 24hr. **f**) Effect of compounds 46-57 on CD147 glycosylation in Hela for 24hr. **g**) Effect of compounds 58-89 on CD147 glycosylation in Hela for 24hr. **h**) Effect of compounds 65-76 on CD147 glycosylation in Hela for 24hr. **i**) Effect of compounds 77-88 on CD147 glycosylation in Hela for 24hr.

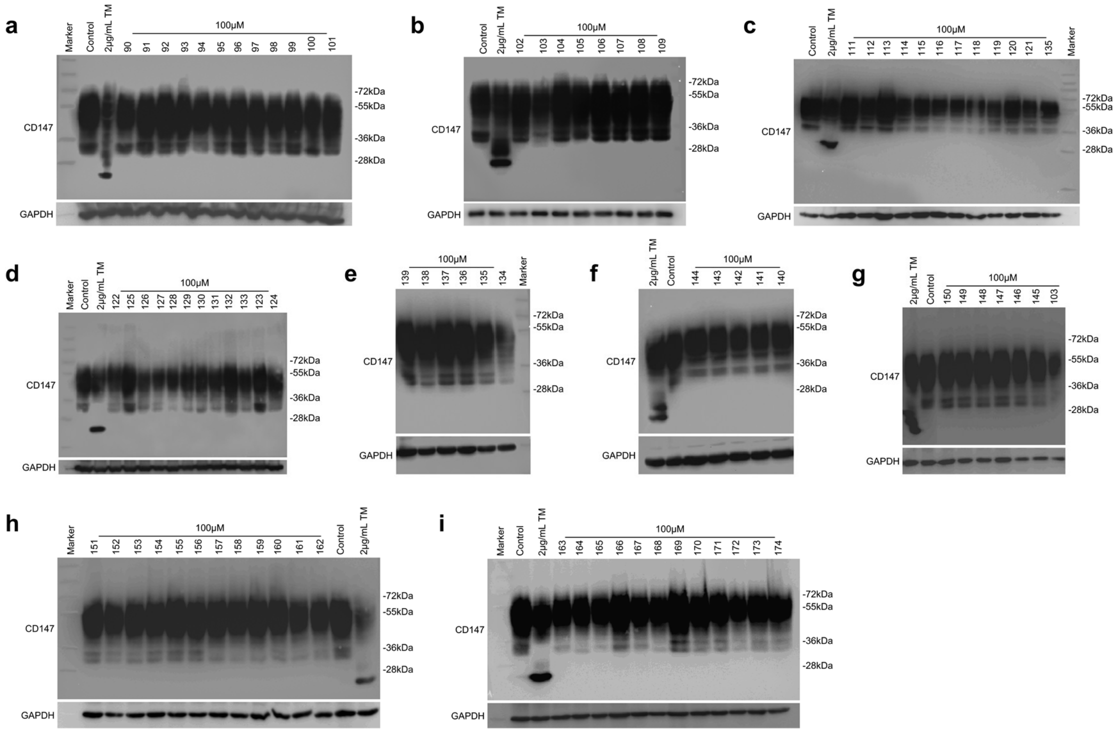

Figure S2. The preliminary screening of glycosylation inhibitors. **a**) Effect of compounds 90-101 on CD147 glycosylation in Hela for 24hr. **b**) Effect of compounds 102-109 on CD147 glycosylation in Hela for 24hr. **c**) Effect of compounds 111-121 and 135 on CD147 glycosylation in Hela for 24hr. **d**) Effect of compounds 122-133 on CD147 glycosylation in Hela for 24hr. **e**) Effect of compounds 134-139 on CD147 glycosylation in Hela for 24hr. **f**) Effect of compounds 140-144 on CD147 glycosylation in Hela for 24hr. **g**) Effect of compounds 145-150 and 103 on CD147 glycosylation in Hela for 24hr. **h**) Effect of compounds 151-162 on CD147 glycosylation in Hela for 24hr. **i**) Effect of compounds 163-174 on CD147 glycosylation in Hela for 24hr.

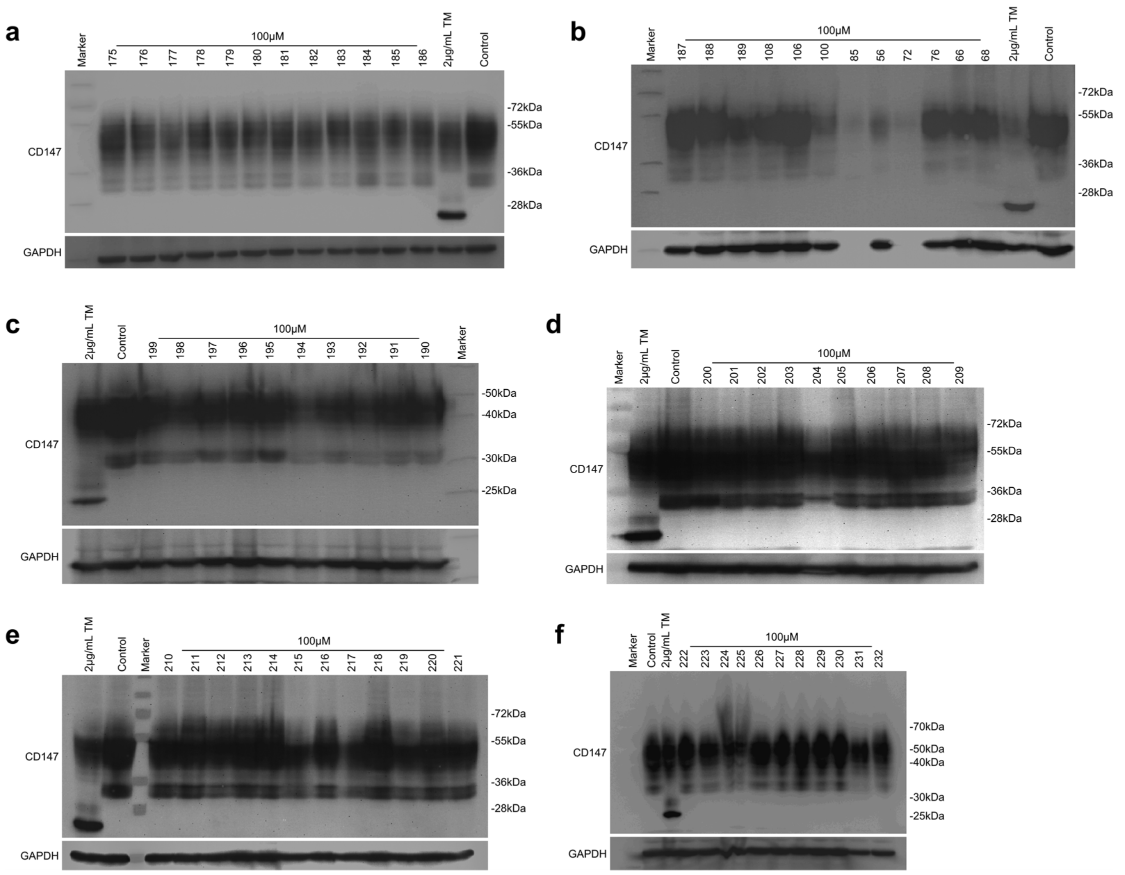

Figure S3. The preliminary screening of glycosylation inhibitors. **a**) Effect of compounds 175-186 on CD147 glycosylation in Hela for 24hr. **b**) Effect of compounds 187-189, 108, 106, 100, 85, 56, 72, 76, 66, and 68 on CD147 glycosylation in Hela for 24hr. **c**) Effect of compounds 190-199 on CD147 glycosylation in Hela for 24hr. **d**) Effect of compounds 200-209 on CD147 glycosylation in Hela for 24hr. **e**) Effect of compounds 210-221 on CD147 glycosylation in Hela for 24hr. **f**) Effect of compounds 222-232 on CD147 glycosylation in Hela for 24hr.

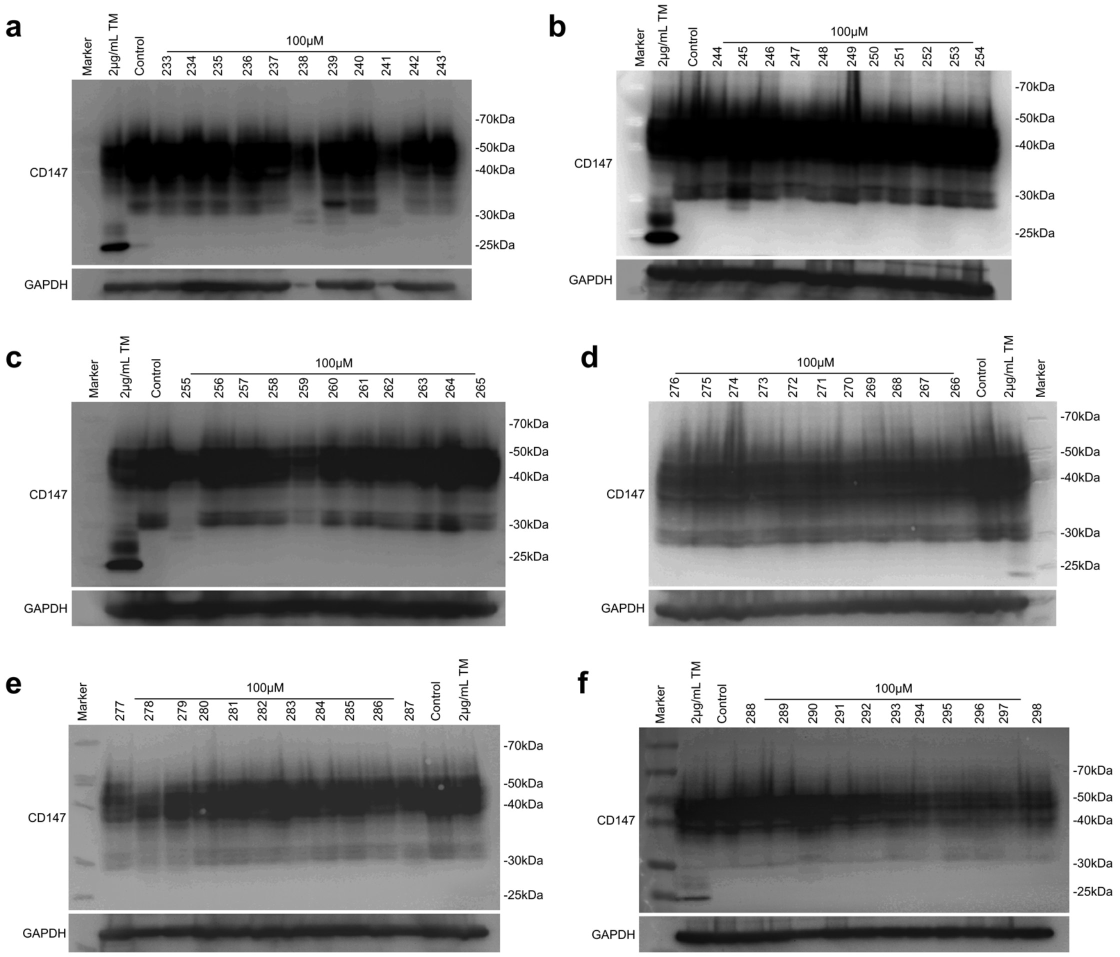

Figure S4. The preliminary screening of glycosylation inhibitors. **a**) Effect of compounds 233-243 on CD147 glycosylation in Hela for 24hr. **b**) Effect of compounds 244-254 on CD147 glycosylation in Hela for 24hr. **c**) Effect of compounds 255-265 on CD147 glycosylation in Hela for 24hr. **d**) Effect of compounds 266-276 on CD147 glycosylation in Hela for 24hr. **e**) Effect of compounds 277-287 on CD147 glycosylation in Hela for 24hr. **f**) Effect of compounds 288-298 on CD147 glycosylation in Hela for 24hr.

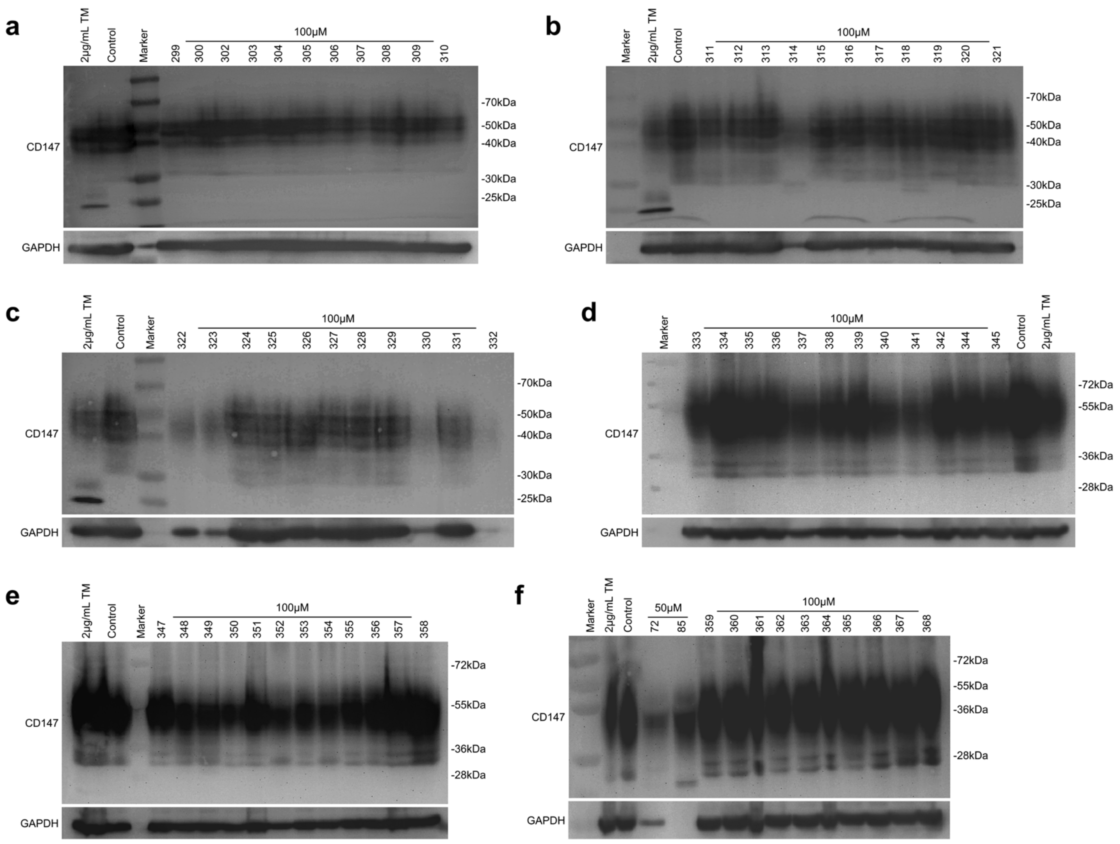

Figure S5. The preliminary screening of glycosylation inhibitors. **a**) Effect of compounds 299-300 and 302-310 on CD147 glycosylation in Hela for 24hr. **b**) Effect of compounds 311-321 on CD147 glycosylation in Hela for 24hr. **c**) Effect of compounds 322-323 on CD147 glycosylation in Hela for 24hr. **d**) Effect of compounds 333-345 on CD147 glycosylation in Hela for 24hr. **e**) Effect of compounds 347-358 on CD147 glycosylation in Hela for 24hr. **f**) Effect of compounds 72, 85, and 359-368 on CD147 glycosylation in Hela for 24hr.

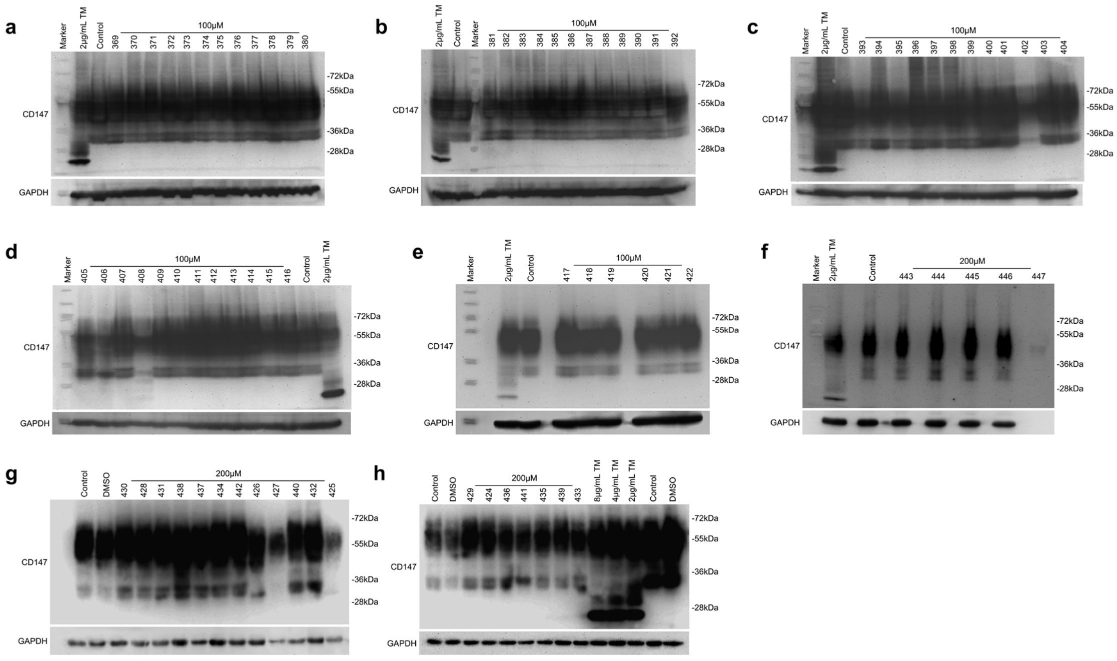

Figure S6. The preliminary screening of glycosylation inhibitors. **a**) Effect of compounds 369-380 on CD147 glycosylation in Hela for 24hr. **b**) Effect of compounds 381-392 on CD147 glycosylation in Hela for 24hr. **c**) Effect of compounds 393-404 on CD147 glycosylation in Hela for 24hr. **d**) Effect of compounds 405-416 on CD147 glycosylation in Hela for 24hr. **e**) Effect of compounds 417-422 on CD147 glycosylation in Hela for 24hr. **f**) Effect of compounds 443-447 on CD147 glycosylation in Hela for 24hr. **g**) Effect of compounds 1425-428, 430-432, 434, 437-438, 440, and 442 on CD147 glycosylation in Hela for 24hr. **h**) Effect of compounds 429, 433, 435-436, 439, and 441 on CD147 glycosylation in Hela for 24hr.

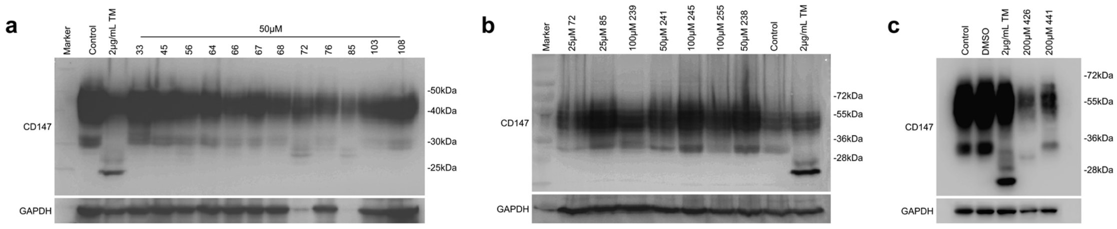

Figure S7. A repeat of glycosylation inhibition assays for the potential compounds. **a**) Effect of compounds 33, 45, 56, 64, 66, 67, 68, 72, 76, 85, 103, and 108 on CD147 glycosylation in Hela for 24hr. **b**) Effect of compounds 72, 85, 239, 241, 245, 255, and 238 on CD147 glycosylation in Hela for 24hr. **c**) Effect of compounds 426 and 441 on CD147 glycosylation in Hela for 24hr.

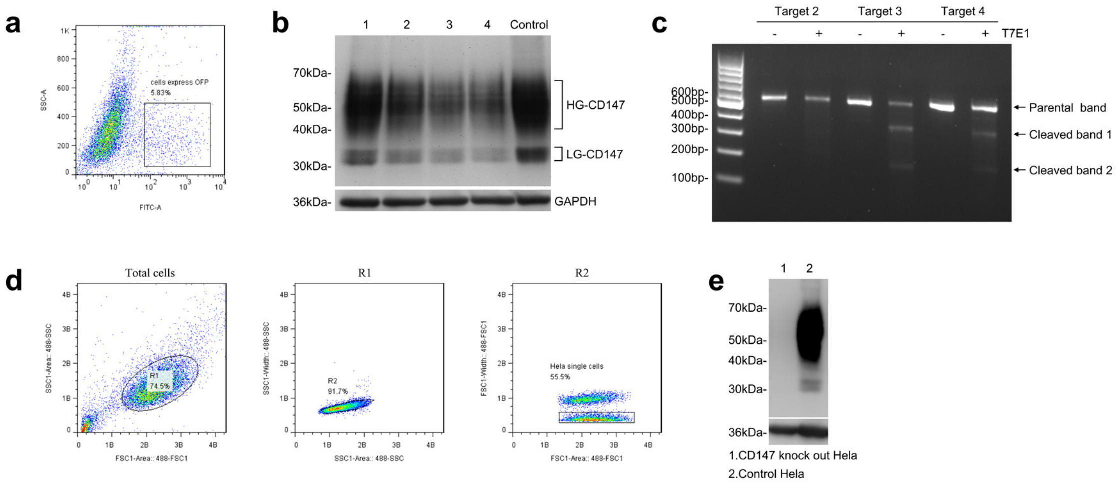

Figure S8. Construction of CD147 knockout Hela cell line. **a**) Enrichment of CRISPR nuclease expressing cells which expressed reporter gene OFP. **b**) Detection of editing efficiency of four guide RNA sequences (1, 2, 3, 4) targeting CD147. The editing of target 2 and 3 in the CD147 locus can effectively reduce the expression of CD147 protein. **c**) Genomic cleavage detection near the editing site 2, 3, and 4 in the CD147 locus by T7E1. **d**) Sorting of CRISPR nuclease expressing single cell containing editing sites 3 or 4. **e**) Protein levels of CD147 were examined in total lysates of Hela cells and Hela CD147 knockout single cell clone.
